# Biological Rationale and Clinical Evidence of Carbon Ion Radiation Therapy for Adenoid Cystic Carcinoma: A Narrative Review

**DOI:** 10.3389/fonc.2021.789079

**Published:** 2021-11-30

**Authors:** Pierre Loap, Barbara Vischioni, Maria Bonora, Rossana Ingargiola, Sara Ronchi, Viviana Vitolo, Amelia Barcellini, Lucia Goanta, Ludovic De Marzi, Remi Dendale, Roberto Pacelli, Laura Locati, Valentin Calugaru, Hamid Mammar, Stefano Cavalieri, Youlia Kirova, Ester Orlandi

**Affiliations:** ^1^Radiation Oncology Unit, Clinical Department, National Center for Oncological Hadrontherapy (CNAO), Pavia, Italy; ^2^Department of Radiation Oncology, Institut Curie, Paris, France; ^3^Proton Therapy Center, Institut Curie, Orsay, France; ^4^Department of Advanced Biomedical Sciences, University of Naples “Federico II”, Napoli, Italy; ^5^Institut Curie, PSL Research University, University Paris Saclay, INSERM LITO, Orsay, France; ^6^Medical Oncology Department, Fondazione IRCCS Istituto Nazionale dei Tumori, Milan, Italy; ^7^Department of Oncology and Hemato-Oncology, University of Milan, Milan, Italy

**Keywords:** adenoid cyst carcinoma, hadrontherapy, carbon ion radiotherapy (CIRT), tumor immunology, radioresistance

## Abstract

Adenoid cystic carcinoma (ACC) is a rare, basaloid, epithelial tumor, arising mostly from salivary glands. Radiation therapy can be employed as a single modality for unresectable tumors, in an adjuvant setting after uncomplete resection, in case of high-risk pathological features, or for recurrent tumors. Due to ACC intrinsic radioresistance, high linear energy transfer (LET) radiotherapy techniques have been evaluated for ACC irradiation: while fast neutron therapy has now been abandoned due to toxicity concerns, charged particle beams such as protons and carbon ions are at present the beams used for hadron therapy. Carbon ion radiation therapy (CIRT) is currently increasingly used for ACC irradiation. The aim of this review is to describe the immunological, molecular and clinicopathological bases that support ACC treatment with CIRT, as well as to expose the current clinical evidence that reveal the advantages of using CIRT for treating ACC.

## 1 Introduction

Adenoid cystic carcinoma (ACC) is a rare tumor, which has a dual component of myoepithelial and ductal cells. Most of the ACCs arise from minor salivary glands, and account for about 10% of all malignant salivary tumors (1). Other involved head and neck (HN) sites include the external ear and the lacrimal glands. Less frequently, ACC might be diagnosed in non-HN sites, including the breasts, the lungs, the prostate gland, the esophagus, the cervix, the vulva or the skin (2–4). Overall, around 3000 cases have been identified between 1973 and 2007 in the US National Cancer Institute’s Surveillance, Epidemiology and End Results (SEER) program (5) and around 2600 cases have been described between 1983 and 1994 in the European EUROCARE registry (6).

Photon beam radiotherapy (RT) technological developments gave a substantial contribution in HN patient prognosis improvement last decades especially in patients bearing squamous cell carcinoma (7). RT plays a key role in different phases of ACC management: in the adjuvant setting after surgery for potentially resectable cases or as a definitive modality for non-operable tumors; or in a reirradiation context for local recurrent disease (8). Due to the radioresistance of ACC, RT techniques using high linear energy transfer (LET) particles have been evaluated for more than 50 years, in particular fast neutron beams (9). However, the use of neutron beams, while efficient, has been abandoned for late toxicity due to the difficulty of obtaining an advantageous dose gradient between the target and the organs-at-risk. On the other hand, carbon ion radiation therapy (CIRT) harbors both the high LET of fast neutron beams and the specific spatial distribution of charged particle beams. Charged particle beams are characterized by a Bragg peak that deposits most of the dose in a very short path, the deepness of which depends on the start energy of the beam, allowing targeted irradiation. CIRT has consequently been increasingly evaluated for ACC irradiation during the last years.

To this date, no *in vitro* or preclinical study have specifically evaluated CIRT irradiation effects on ACC cell lines. Nevertheless, multiple immunological and biological properties justify ACC management with CIRT (**Table 1**). We reviewed the literature on the biological bases and the clinical evidence that support the use of CIRT in ACC management.

**Table 1:** Biological rationale for adenoid cystic carcinoma (ACC) with carbon ion radiation therapy (CIRT).

ACC adverse characteristics	Molecular determinants	Biological rationale of CIRT
Tumor antigenicity	Low TMB	↗ tumor immunogenicity
Immunosurveillance escape	↗ PD-L2 and HLA-G expression	↗ ICAM1
	↘ ICAM-1 expression	
Immunotolerant microenvironment	↘ CD1a and CD83 infiltrate	↗ DC
	↘ MDSC and M2 macrophage infiltrate	↘ M2 and MDSC
	T-cell exclusion phenotype	↗ proinflamattory cytokines
		↗ CD8, ± NK
Hypoxia	↗ HIF1a expression	low OER
	VEGFA-mediated vascular mimicry	↘ tumorigenesis and angiogenesis
Stemness	↗ HSP27 expression	Anti-tumor response on radioresistant tumor cell lines
	↗ Brachyury expression	
	VEGF A, Nodal, Lefty, Oct-4, Pac6, Rex1, Nanog	
Autophagy	ATG3, 4A, 5, PIK3R4, MAP1LC3B	
Perineural invasion	BNDF/TrkB; CCLR/CCR5; NGF/TrkA	↘ migration, invasion, adhesion
		↘ cell mobility
		↘ integrin expression
Tumoral heterogeneity	Biphasic tumor: ductal and myoepithelial components	Anti-tumor response ± independent on tumoral heterogeneity
	Molecular heterogeneity within/between primary tumors and metastatic disease	

The rationale to use CIRT for ACC management is based on immunological, molecular, and pathological considerations, despite the fact that no *in vitro* or preclinical study have specifically evaluated CIRT irradiation on ACC cell lines; CD, cluster of differentiation; DC, dendritic cell; HIF1a, hypoxia-inducible factor 1a; ICAM-1, intercellular adhesion molecule 1; MDSC, myeloid-derived suppressor cell; NK, natural killer cell; OER, oxygen enhancement ratio; TMB, tumor mutational burden; VEGF, vascular-endothelial growth factor.

## 2 Molecular Rationale of CIRT for ACC

### 2.1 ACC Heterogeneity Represents a Challenge for Radiotherapy

#### 2.1.1 ACC Is a Heterogeneous Group of Tumors From a Molecular Point of View

Based on whole-exome sequencing (WES) of 34 tumor samples from primary and metastatic ACC tumors isolated from eight patients, Liu et al. (10) demonstrated that there was an important spatial and temporal clonal diversity within and between primary and metastatic tumors. The average mutation rate was evaluated around 0.32 per million base pair and the incidence of shared mutations between primary and metastatic tumor samples was 21.9%; truncal genetic alterations included NOTCH pathway genes (such as NOTCH1 or SPEN) or the t (6, 9) translocation (MYB–NFIB fusion). Nevertheless, this apparent diversity of tumor mutations allows a variety of potential systemic targeted treatments. For instance, Ho et al. (11) demonstrated that tumoral activation of the VEGF/KIT/PDGFR pathway could be effectively targeted by anti-angiogenic agents (such as Axitinib), while the NOTCH-mutated ACCs could be specifically blocked by agents targeted NOTCH pathway (such as Bronctictuzumab or AL101) (12, 13). NOTCH-mutated ACC are over-represented in solid variant and are associated with a higher rate of liver and bone metastases, as well as shorter relapse-free survival (RFS) and overall survival (OS) (12). The molecular diversity of ACC tumors is striking in a relapsed or a metastatic context (14). Up to 26.3% of relapsed or metastatic ACC patients are NOTCH-mutated, including 18.3% of activating NOTCH mutations, which have the poorest prognosis among all NOTCH mutations types. Ho et al. (14) further demonstrated that mutations in the KDM6A gene, which intervenes in chromatin remodeling, had a pejorative prognosis for relapsed or metastatic ACC, and that TERT pathway mutations were exclusive of NOTCH mutations and of MYB fusions. Consequently, it appeared that four distinct relapsed/metastatic ACC molecular subgroups could be proposed, based on the presence of NOTCH, TERT mutations and MYB fusion: MYB-mutated/NOTCH-mutated; MYB-mutated/other mutations; MYB wild-type/NOTCH-mutated and MYB-mutated/TERT-mutated.

#### 2.1.2 ACC Is a Heterogeneous Group of Tumors From an Anatomopathological Point of View

ACC is characterized by a biphasic composition made of ductal cells (characterized by CK7 protein expression) and myoepithelial cells (characterized by CK5/6, P63, P40, D2-40, Calponin, a-SMA, S-100, and vimentin protein expression). The histological tumoral organization defines three ACC variants based on the predominant anatomopathological pattern: cribriform, tubular (both characterized by CK7 protein expression), or solid pattern (characterized by a glandular architecture and a loss of myoepithelial differentiation). Solid patterns usually have a higher Ki67 index. Multiple histological grading systems have been proposed based on the estimated proportion of the solid pattern component [Perzin grading using a 30% threshold (15), Spiro grading using a 50% threshold (16)]. It should be noted, however, that Van Weert et al. (17) recently demonstrated that the mere presence of solid components, independently of its proportion, was a negative prognostic factor. Bell et al. (18) evidenced that the c-Kit protein was systematically expressed, and EGFR was consistently negative, in the solid ACC subtype. C-Kit expression was limited to inner ductal epithelial cells, and EGFR expression was restrained to the outer myoepithelial cells, the latter being found in the majority of tubular and cribriform ACC patterns.

#### 2.1.3 CIRT Anti-Tumor Efficiency Is Not Influenced by Tumor Heterogeneity

Based on three cellular sublines of resistant prostate tumors in murine models, Glowa et al. (19) demonstrated that the values of the tumor control dose 50 (TCD50) differed significantly less for CIRT than for photon RT. They concluded that response to CIRT was relatively independent on the molecular and histological tumoral heterogeneity. Additionally, Masunaga et al. (20) found that quiescent tumor cells were more sensitive to CIRT than to photon RT. They suggested that CIRT anti-tumor efficacy could be relatively independent from intra-tumoral cellular heterogeneity, resulting from the co-existence of quiescent and proliferative cell populations in various proportions.

### 2.2 ACC Radioresistance Properties Might Be Overcome by CIRT

ACC are radioresistant tumors. The main molecular actors of ACC EMT and radioresistance features are provided in **Table 2**.

**Table 2:** Notable molecular actors of ACC stemness and radioresistance properties.

Molecular actor		Roles in ACC radioresistance
**BNIP3**	BCL2/adenovirus E1B 19 kDa protine-interacting protein 3	Apoptotic Bcl-2 protein. Intervenes in autophagosome formation; can induce autophagic cell death.
**Brachyury**		Transcription factor. Represses expression of adhesion molecules which promotes epithelial mesenchymal transition (EMT)
**CD133/CD44**		CD133: Surface glycoproteine. Marker of cancer stem cells.
** **		CD44: Surface glycoprotein. Intervenes in cellular interactions and cell adhesion
**HIF1α**	Hypoxia-inducible factor 1-alpha	Transcription factor (subunit) responsive to oxygen level. Induces cell proliferation and survival
**HSP27**	Heat Shock Protein 27	Chaperone protein. Has an anti-apoptotic role and a cytoprotection function under stress conditions; modulates reactive oxygen species
**MAP1LC3B**	Microtubule-associated proteins 1A/1B light chain 3B	Ubiquitin-like protein. Selects substrate for autophagic degradation
**Snail**	Zinc finger protein SNAI1	Transcription factor. Represses expression of adhesion molecules which promotes EMT

#### 2.2.1 Epithelial-Mesenchymal Transition and Cancer Stem Cell Properties Are Related to ACC Radioresistance

Wang et al. (21) demonstrated that hypoxic conditions promoted ACC epithelial-mesenchymal transition (EMT) and cancer stem cell (CSC) properties. Molecular actors of ACC EMT and stemness were characterized by Chen et al. (22) who found that HSP27 protein overexpression increased cell migration and invasion properties and induced an up-regulation of Snail 1 and Prrx1, which are potent EMT regulators. Expectedly, increased HSP27 levels in ACC correlated with radioresistance of ACC cell lines *in vitro*; it was also found that high HSP27 expression in ACC had a poor prognosis value. Finally, acquisition of CSC properties in ACC correlated with increased expression of CD133 and CD44. Shimoda et al. (23) further evidenced stemness was a generic characteristic of metastatic ACC cells, which express stemness-related transcription factors (TF) (such as Nodal, Lefty, Oct-4, Pac6, Rex1, and Nanog). In particular, the T-box Brachyury TF was highly expressed in clinical ACC samples and was found to regulate both EMT and CSC properties of ACC. Kobayashi et al. (24) further demonstrated that a knock-out of the Brachyury gene in ACC cells lines with short hairpin RNA (shRNA) suppressed both tumor chemoresistance and radioresistance *in vitro*.

#### 2.2.2 ACCs Are Characterized by Hypoxia Markers

De Mendoça et al. (25) demonstrated that ACCs were associated with high Hypoxia-Inducible Factor (HIF) 1α expression levels compared with normal salivary gland, which is expressed in the absence of adequate tissular oxygenation. Liu et al. (26) further observed that salivary ACC had an up-regulation of autophagy-related genes (such as ATG3, 4A, 5, PIK3R4, and MAP1LC3B), controlled by HIF1α. It should be recalled that hypoxia-induced autophagy is a notable actor of resistance to anti-tumor treatments, including radiation therapy (27). Chen et al. (28) demonstrated that BNIP3, a regulator of hypoxia-induced autophagy, was expressed by 63.1% of ACCs, and that BNIP3 in ACC cell lines could be induced *in vitro* by hypoxia. Another notable consequence of ACC hypoxia was to induce tumoral vascular mimicry and cell migration invasion as a response to VEGF-A secretion (21, 29).

#### 2.2.3 CIRT Is Valuable for Radioresistant and Hypoxic Tumors

Peschke et al. (30) found on murine models of radioresistant prostate carcinomas that the TCD50 were 32.9 Gy for CIRT and 75.7 Gy for photon RT for single dose irradiation, and 38.0 Gy for CIRT and 90.6 Gy for photon radiotherapy for multiple-dose irradiation. This observation suggested that CIRT was more potent than photon radiotherapy for intrinsically radioresistant tumors, which is the case for ACC. In addition, Grimes et al. (31) evidenced that carbon ion beams had a lower oxygen enhancement ratio (OER) than proton beams, in particular towards the Bragg peak where the LET substantially increased for carbon beams, making CIRT valuable in case of hypoxic tumors. Nevertheless, Antonovic et al. (32) underlined that hypoxia could anyhow influence the outcome of CIRT because of the non-negligible OER of the low LET contributions in the spread-out Bragg peak (SOBP). Taking into account inter-fraction local oxygenation changes, occurring after tumor shrinkage (even for hypoxic tumors), CIRT OER was estimated around 1.2. Finally, using a glioma model, Liu et al. (33) demonstrated that CIRT superiority in tumorigenesis and angiogenesis inhibition compared with photon beams, resulted from modulation of VEGF level in the tumor micro-environment (TME).

### 2.3 CIRT May Control ACC Tumoral Invasion Properties

#### 2.3.1 Perineural Invasion Is a Characteristic Feature of ACC Local Malignancy

ACCs are characterized by an elevated propensity to locally invade surrounding tissues through perineural invasion (PNI). Shan et al. (34) demonstrated that the BDNF/TrkB axis plays a causative role in ACC PNI. Gao et al. (35) found that the CCL5/CCR5 axis increases salivary ACC PNI invasion and that blocking this chemokine axis inhibited perineural invasion in ACC cell lines. CCR5 chemokine receptor expression was elevated in salivary ACC tissue samples. Kobayashi et al. (36) observed that NGF and TrkA signaling contributed to PNI, and that both were expressed in around 65% of ACC patients.

#### 2.3.2 CIRT Might Control Tumoral Invasion

Fujita et al. (37) demonstrated that CIRT irradiation decreased tumor cell mobility through a process involving ubiquitinoylation and proteasome destruction of Rac1 and RhoA proteins. Rieken et al. (38) also found that CIRT decreased tumor integrin expression 24h after irradiation, and significantly reduced glioma cell migration. Matsumoto et al. (39) observed that CIRT could reduce the metastatic abilities of malignant melanoma cells (including cell migration, invasion, and adhesion), both *in vitro* and *in vivo*, in murine models.

## 3 Immunological Rationale of CIRT for ACC

### 3.1 ACC Tumoral Immunology

#### 3.1.1 ACC Cell Immunogenicity Is Limited to Few Tumor-Associated Antigens

ACC cells express few tumor-specific neo-antigens (TNA) against which specific anti-tumor immune responses could be directed, which is explained by their low tumoral mutation burden (TMB). Based on 60 ACC tumor samples, Ho et al. (40) estimated that ACC TMB was around 0.31 non-silent mutation per megabase. Nevertheless, TMB-high ACCs have already been described, either microsatellite-instable (MSI) or POLE-mutated, but these cases represent only a minority of ACC patients (41). Consequently, T-cell receptor (TCR) clonotype diversity in ACCs is usually lower than in most other solid tumor types and CD8 tumor-infiltrating lymphocytes (TIL) are rare (41). On the other hand, ACC cells express diverse types of tumor-associated antigens (TAA), in particular cancer-testis antigen (CTA). Based on 84 head and neck (HN) ACC tumor samples, Veit et al. (42) found that NY-ESO-1 and pan-MAGE CTA were significantly expressed in 57.1% and 31.2% of ACC patients, respectively. In addition, tumor expression of these two CTA has been found to be linked to a worse prognosis, since median overall survival (OS) was 282 months in the absence of NY-ESO-1 and pan-MAGE expression, 190.5 months when one of these two antigens was present, and only 90.5 months in case of simultaneous co-expression. Beppu et al. (43) estimated that MAGE-A CTA was detected in 60% of ACC tumors and represented an independent risk factor for locoregional recurrence. Finally, in addition to CTA, ACC also expresses less immunogenic tissue-differentiation TAA. Prostate-specific membrane antigen (PSMA) expression without predictive value was found in 94% of ACC patients (44).

#### 3.1.2 ACC Cells Evade Immune Surveillance by Regulating the Expression of Membrane Receptors

Based on immunohistochemistry (IHC) analyses on 36 ACC tumor samples, Mosconi et al. (45) found that ACC expressed a high level of inhibitory immune membrane proteins, particularly PD-L2 and HLA-G. Notably, PD-L1 expression was systematically negative, and CTLA-4 expression was low, which are the targets of most current immune checkpoint inhibitors. PD-L2 expression has a prognosis value: Chang et al. (46) demonstrated that low PD-L2 expression was associated with a shorter RFS in a cohort of 70 patients with malignant salivary gland tumors (including 15 ACC). In addition, ACC cells have a reduced expression of ICAM-1 adhesion protein (47), and IHC analysis of tumor samples demonstrated reduced staining for surface antigens of T cells, NK cells, macrophage (TIA1 and CD68) (47), and antigen-presenting cells (APC) (CD1a and CD83) (45). It has consequently been proposed that the reduced ACC membrane expression of ICAM-1 might promote immune evasion by limiting the ICAM-1/LFA-1-mediated interaction between ACC cells and anti-tumoral immune cells. Conversely, high ICAM-1 expression was associated with a significantly better DFS for ACC (47).

#### 3.1.3 ACC Tumoral Micro-Environment Is Characterized by a Pro-Tumoral Immune Polarization

ACC TME is characterized by a pro-tumoral polarization. Based on WES of 76 malignant salivary tumor samples, including ACC, myoepithelial carcinomas (MECA) and salivary duct carcinomas (SDC), Linxweiler et al. (48) demonstrated that ACC had the highest infiltration of M2-polarized tumor-associated macrophages (TAM) and of myeloid-derived suppressor cells (MDSC), among all types of salivary gland carcinomas. Simultaneously, ACC TME had the lowest infiltration of anti-tumoral immune cells, with a T-cell exclusion phenotype characterized by a limited population of innate immunity cells (NK cells, mast cell, neutrophils, macrophages, and eosinophils), of APC [dendritic cells (DC)], and lymphocytes (CD8 T cells; Th1, Th2, and Th17 CD4 T cells; follicular helper T cells; regulator T cells; γδ T cells; and B cells). Sridharan et al. (49) demonstrated that alterations in the PI3K and the WNT pathways (in particular, involving FGF17, BCL2, beta-catenin, and BAMBI genes) strongly correlated with a lack of immune-cell infiltrates in ACC TME. In addition, it was found that cytokine landscape of ACC TME contributed to its pro-tumoral properties: CCL2 chemokine produced by ACC cells recruit M2-polarized TAM, which, in response, increase tumor cell invasive and migrative properties by secreting glial cell line-derived neurotrophic factor (GDNF).

### 3.2 CIRT and Immune System Modulation

#### 3.2.1 CIRT Increases Tumoral Cell Immunogenicity

Imadome et al. (50) found that CIRT upregulated stress-responsive genes and immunity-related cell-communication genes (notably ICAM1) in tumor cells. At the same time, CIRT increased gene expression of cytokines and chemokines. Six to 36 hours after CIRT irradiation, Ohkubo et al. (51) demonstrated that an increased expression of ICAM-1 membrane receptor could be observed, which interacts with APC through their LFA1 receptors, but which is usually downregulated in ACC tumors (47). Based on murine lung tumor models, it was demonstrated that combination of CIRT with DC inhibited the development of lung metastases (51), while Ando et al. (52) evidenced that CIRT increased tumor cells immunogenicity and DC activation to a higher level than photon beam radiotherapy, as evidenced by greater CD40 and IL-12 level.

#### 3.2.2 CIRT Activates Adaptative Immunity

Hartman et al. (53) found *in vitro* that CIRT and photon beam radiotherapy had common radiobiological properties such as induction of cell cycle arrest, surface expression of immune-modulating molecules, and activation of cytotoxic lymphocytes. Nevertheless, other authors have suggested that CIRT-induced immune activation might be more potent. Spina et al. (54) demonstrated that CIRT could induce a more pro-inflammatory cytokine landscape compared with photon beam radiotherapy on mammary tumor cell lines: high levels of IL-2, IL-1β, and IFNγ were observed after CIRT (compared with an isolated IL-6 increase after photon radiotherapy). Simultaneously, CIRT induced an activated CD8 TIL phenotype in TME, as evidenced by high levels of granzyme B, IL-2, and TNFα expression, while the authors found that photon therapy decreased CD8 TILs. Takahashi et al. (55) demonstrated that, when combined with anti-PD-L1 and anti-CTLA-4 immunotherapies, CIRT substantially increased CD8 TIL infiltrates and HMGB-1 level, a potent danger-associated molecular pattern (DAMP), suggesting a rational therapeutic strategy combining immune checkpoint inhibitors with CIRT.

#### 3.2.3 TME Acquires Anti-Tumoral Polarization After CIRT Irradiation

In a murine glioblastoma model, Chiblak et al. (56) demonstrated that CIRT reduced the population of M2-polarized TAM and MDSC while simultaneously increasing CD8 TILs, compared with photon beam radiotherapy. Xie et al. (57) further evidenced that low-dose whole-body CIRT of mice (up to 0.05Gy) might increase NK cell activity and induce an IFNγ pro-inflammatory cytokine signature. The propensity of CIRT to induce an immune-permissive anti-tumoral TME might be of significant interest for ACC management.

## 4 Clinical Considerations on ACC Irradiation With CIRT

### 4.1 Photon RT Leads to Substantial Toxicity Due to Critical OAR Radiation Exposure

Photon radiotherapy for ACC management usually leads to unsatisfying exposure of organs-at-risk (OAR) with substantial toxicity. On the other hand, CIRT, taking advantage of a superior dose deposition characterized by a Bragg peak, increases OAR sparing.

#### 4.1.1 OAR Toxicity for Head and Neck ACC Treated With Photon RT

Münter et al. (58) irradiated with intensity-modulated radiation therapy (IMRT) 17 ACC localized at the base of the skull or in maxillary sinuses with a median dose of 66Gy observed 20% of grade 3 mucositis. For sinonasal tumors (including 4 ACC patients) treated to the median dose of 70 Gy with IMRT, Madani et al. (59) also found non-negligible radiation-induced toxicity with 14.1% of grade 3 mucositis, 5.1% of grade 3 dysphagia, 5.1% of grade 3 dermatitis, and three cases of asymptomatic radio-necrosis. Lesueur et al. (60) evaluated that lachrymal ACC radiation therapy could be associated with brain radio-necrosis, bone exposure, radiation neuropathy, secondary glaucoma, and radiation neuropathy.

#### 4.1.2 OAR Toxicity for Thoracic ACC Treated With Photon RT

Thoracic ACC RT exposes multiple OARs: the heart, the lungs, the esophagus, the trachea, and the thyroid. Out of 31 tracheal ACC patients treated with photon RT (mean dose of 62 Gy), Levy et al. (61) observed five tracheal stenoses, four dyspnea, five hypothyroidism, and four pericarditis. Je et al. (62) irradiated 13 tracheal ACC patients with photon RT (59.4 Gy in an adjuvant setting and 74.4 Gy in a definitive setting): two patients developed tracheal stenoses, and both died (abrupt respiratory failure after 1 year and tracheal infection after 13 years). Finally, Dracham et al. (63) treated 19 tracheal ACC patients with photon RT (50 Gy for adjuvant and 67.8 Gy for definitive setting) and observed seven grade ≥2 acute pneumonitis and two grade 3 esophagitis.

#### 4.1.3 OAR Toxicity for Digestive and Pelvic ACC Treated With Photon RT

The largest cohort of Bartholin’s gland ACC has been reported by Cardosi et al. (2) consisting of 12 patients treated with surgery; seven patients underwent adjuvant RT. One vulva radio-necrosis was observed, followed by a grade 5 sepsis, and one patient developed concomitant digestive and urinary fistulas. It should be recalled that fistulas have a significant impact on the quality of life of the patient. Zelga et al. (64)demonstrated that the simplest and safest treatment of radiation-induced rectovaginal fistulas was a fecal diversion with an ileostomy. While cardia (65) and esophageal (3) ACC exist, no report of RT-induced toxicity with photon RT has been published to this date; nevertheless, CIRT is expected to reduce radiation exposure to the unaffected digestive tract and to the heart.

### 4.2 Evidence of CIRT by ACC Tumor Site

#### 4.2.1 Materials and Methods

A search was conducted on the PubMed, Medline, Google Scholar, Cochrane library and Web of Science databases using the following keywords: [“particle therapy” or “hadrontherapy” or “carbon ion” or “CIRT” or “heavy ion” or “ion beam” or “ion radiation”] and [“adenoid cystic carcinoma” OR “ACC”]. Search was independently conducted by PL and EO. Inclusion criteria, defined using the PICOS framework, were the following: clinical studies (trials, prospective or retrospectives studies, and case reports) evaluating CIRT for ACC, in any setting, and reporting toxicity and efficacy data. Exclusion criteria were pre-clinical or purely dosimetric studies. References from the selected studies were screened for potential additional articles.

#### 4.2.2 Head and Neck (Primary Tumor)

The current clinical experience of CIRT for ACC is summarized in the **Table 3**. CIRT has been evaluated as a sole modality for definitive irradiation of head and neck ACC by Japanese centers. Mizoe et al. (67) evaluated the efficacy of 64 Gy(RBE) on 236 patients with head and neck cancers, including 69 ACC (mostly from the paranasal sinuses), with 5-year LC and OS of 73% and 68%, respectively. Sulaiman et al. (68) retrospectively described the outcome of all ACC patients treated in the four active CIRT facilities in Japan treated between 2003 and 2014. Overall, 289 ACC patients were treated with a median CIRT dose of 64 Gy(RBE) (ranging between 55.2 Gy(RBE) and 70.4 Gy(RBE)); 2-year OS, PFS, LC were 94%; 68% and 88%, respectively. Two patients died from bleeding ulcers, and 15% of all patients developed grade ≥3 toxicity. Ikawa et al. (71) observed 5-year LC and OS of 78.8% and 58.3% in a cohort of 74 oral non-squamous cell carcinomas, including 34 ACC patients, treated with doses ranging from 57.6 to 64 Gy(RBE). Analyses of specific tumoral localization demonstrated the efficacy of CIRT for ACC arising from the nasopharynx (72) (2-year LC: 88%), from the paranasal sinuses (73)(5-year LC: 51%), from the tongue (74)(5-year LC: 92%), from the parotid (66) (5-year LC: 74.5%), or from the lacrimal gland (69)(5-year LC: 62%).

**Table 3:** Clinical studies evaluating carbon ion radiation therapy (CIRT) for adenoid cystic carcinoma (ACC) irradiation.

Indication	Study	Year	Center	Type	Number	CIRT fractionation	Efficacy	Grade ≥3 toxicity	Grade 4-5 toxicity (detail)
Head and neck (diverse sites)	Schulz-Ertner et al. (66)	2004	Heidelberg	Retrospective	21 ACC (out of 152 tumors)	18 Gy(RBE) CIRT boost + 54 Gy photon RT.	3-year LRC: 62%. 3-year OS: 75%.	Acute: 2 pts. Late: 0 pt (10%). (ACC cohort)	Ø (ACC cohort)
	Mizoe et al. (64)	2011	NIRS	Phase II	69 ACC (out of 236 tumors)	57.6-64 Gy(RBE)/16 fr	5-year LC: 73%. 5-year OS: 68%.	Acute: 39 pts (56%). Late: 4 pts (6%).	4 G4 blindness (late).
	Sulaiman et al. (65)	2017	NIRS, Hyogo, Gunma, HIMAT	Retrospective	289 ACC	57.6-64 Gy(RBE)/16 fr	5-year LC: 68%. 5-year OS: 74%.	Acute: 92 pts (32%). Late: 48 pts (17%).	2 G5 hemorrhage, 9 G4 blindnesses, 1 G4 brain necrosis(late).
	Ikawa et al. (67)	2019	NIRS	Retrospective	34 ACC (out of 74 tumors)	57.6-64 Gy(RBE)/16 fr	5-year LC: 75.2%. 5-year OS: 65.7%.	Acute: 43 pts (58%). Late: 22 pts (30%) (10 G3 osteonecrosis). (Whole cohort)	3 G4 blindness (late). (Whole cohort)
Nasopharynx	Abe et al. (68)	2018	NIRS, Hyogo, Gunma, HIMAT	Retrospective	43 ACC	57.6-64 Gy(RBE)/16 fr	2-year LC: 88%. 2-year OS: 84%.	Acute: 14 pts (33%). Late: 9 pts (21%).	2 G5 pharyngeal hemorrhage, 1 G4 blindness (late).
	Akbaba et al. (69)	2019	Heidelberg	Retrospective	59 ACC	18-24Gy(RBE) CIRT boost + 50-56 Gy photon RT.	5-year LC: 49%. 5-year OS: 69%.	Acute: 7 pts (12%). Late: 4 pt (7%).	Ø
Paranasal sinuses	Akbaba et al. (70)	2019	Heidelberg	Retrospective	227 ACC	15-18Gy(RBE) CIRT boost + 48-56 Gy photon RT.	3-year LRC: 79% (primary) - 82% (postoperative). 3-year OS: 64% (primay) - 79% (postoperative).	Acute: 88 pts (39%). Late: 26 pts (11%).	Ø
	Hagiwara et al. (71)	2020	NIRS	Retrospective	22 ACC	57.6-64 Gy(RBE)/16 fr	5-year LC: 51%. 5-year OS: 62.7%.	Acute: 0 pts. Late: 9 pts (41%).	6 G4 blindness, 1 G4 brain necrosis (late).
Tongue	Koto et al. (72)	2016	NIRS	Retrospective	18 ACC	57.6-64 Gy(RBE)/16 fr	5-year LC: 92%. 5-year OS: 72%.	Acute: 10 pts (56%). Late: 3 pts (16.7%).	Ø
Parotid	Koto et al. (73)	2017	NIRS	Retrospective	16 ACC	57.6-64 Gy(RBE)/16 fr	5-year LC: 74.5%. 5-year OS: 70.1%.	Acute: 1 pt (6%). Late: 8 pts (50%).	1 G4 blindess (late).
Lacrimal gland	Hayashi et al. (74)	2018	NIRS	Retrospective	16 ACC (out of 33 tumors)	57.6-64 Gy(RBE)/16 fr	5-year LC: 62%. 5-year OS: 65%.	Acute: 0 pt. Late: 22 pts (67%). (Whole cohort)	12 G4 blindness, 2 G4 brain necrosis (late). (Whole cohort)
	Akbaba et al. (75)	2019	Heidelberg	Retrospective	18 ACC (out of 24 tumors)	18-24Gy(RBE) CIRT boost + 50-54 Gy photon RT.	5-year LC: 90%. 5-year OS: 94%.	Acute: 3 pts (13%). Late: 2 pt (8%). (Whole cohort)	Ø
Larynx	Akbaba et al. (76)	2018	Heidelberg	Retrospective	8 ACC (out of 15 tumors)	18-24Gy(RBE) CIRT boost + 50-54 Gy photon RT.	3-year LRC: 100%. 3-year OS: 100%.	Acute: 4 pts (27%). Late: 0 pt. (Whole cohort)	Ø
Tracheobronchial tree	Chen et al. (77)	2021	Shanghai	Retrospective	18 ACC	66-72.6 Gy(RBE)/22-23 fr	2-year LC: 100%. 2-year OS: 100%.	Acute: 0 pt. Late: 1 pt (6%).	1 G4 tracheal stenosis (late).
	Högerle et al. (78)	2019	Heidelberg	Retrospective	7 ACC treated with CIRT (out of 38 ACC)	24Gy(RBE) CIRT boost + 50-54 Gy photon RT (n=4). 60-63 Gy(RBE) (n=2)	1-year LC: 100%. 1-year OS: 100%.	Acute: 1 pt (14%). Late: 0 pt. (CIRT cohort)	1 G4 stomatitis (acute).
Bartholin’s gland	Bernhardt et al. (79)	2018	Heidelberg	Retrospective	1 ACC	24Gy(RBE) CIRT boost + 50 Gy photon RT	NA	Ø	Ø
Reirradiation (Head and neck, diverse sites)	Jensen et al. (80)	2015	Heidelberg	Retrospective	48 ACC	51 [36-74] Gy(RBE)	1-year LC: 70.3%. 1-year OS: 81.8%.	Acute: 0 pt. Late: 8 pt (6.5%). (Whole cohort)	2 G4 carotid artery haemorrhage (late).
	Held et al. (81)	2019	Heidelberg	Retrospective	124 ACC (out of 229 tumors)	51 [30-66] Gy (RBE)	1-year LC: 60%. 1-year OS: 72%.	Acute: 7 pt. Late: 18 pt. (Whole cohort)	2 G4 laryngeal edema (acute). 2 G4 blindness, 1 brain necrosis, 1 vascular hemorrhage (late). (Whole cohort)
	Hayashi et al. (82)	2019	NIRS	Retrospective	17 ACC (out of 48 tumors)	54 [40-64] Gy (RBE)	2-year LC: 40.5%. 2-year OS: 59.6%.	Acute: 4 pt. Late: 25 pt. (Whole cohort)	1 G5 brain necrosis, 9 G4 blindness, 1 G4 brain necrosis, 1 G4 infection, 1 G4 arterial injury (late). (Whole cohort)
	Vischioni et al. (83)	2020	CNAO	Retrospective	38 ACC (out of 51 tumors)	60 [45-68.8] Gy(RBE)	2-year PFS: 52.2%. 2-year OS: 64%.	Acute: 2 pt (3.9%). Late: 3 pt (17.5%). (Whole cohort)	Ø

G, grade; LC, local control; LRC, locoregional control; OS, overall survival; PFS, progression-free survival; pts, patients.

An alternative approach has been evaluated at the Heidelberg Ion-Beam Therapy Center, using CIRT as a boost to conventional photon radiotherapy. In 2004, Schulz-Ertner et al. (70) obtained a 3-year LC of 62% on a series of 21 patients affected by head and neck unfavorable and locally advanced ACC, using an 18 Gy(RBE) CIRT boost coupled to photon radiotherapy delivering 54 Gy. Similarly, using CIRT as an 18-24 Gy(RBE) boost to IMRT photon radiotherapy (50-56Gy), Akbaba et al. (76) treated 59 nasopharyngeal ACC patients to a 2-year LC, distant PFS and OS of 83% 81% and 87%. Seven patients developed acute grade 3 toxicities (mucositis, dysphagia, and odynophagia) and four patients had late grade 3 toxicities (locked jaw, tympanic effusion, and hypopituitarism). Akbaba et al. (75) treated 227 patients for sinonasal ACC with an 18-24 Gy(RBE) CIRT boost added to IMRT radiotherapy (48-56Gy). With a median follow-up of 50 months, 3-year local control was 79% when treated in a definitive context and 82% when treated in an adjuvant setting. Acute toxicity was observed for 34.4% of the patients in a definitive setting and for 41.6% in an adjuvant setting. CIRT as a boost to IMRT has also been evaluated for laryngeal ACC (80), demonstrating an excellent local control on a cohort of eight ACC patients without any relapse at 24 months. Lacrimal ACC has also been treated with a CIRT boost (81), with a 2-year local control of 93%.

#### 4.2.3 Head and Neck (Reirradiation)

Jensen et al. (82) re-irradiated 52 recurrent head and neck ACC with a median dose of 51 Gy(RBE). Median follow-up was 14 months; one-year local and distant controls were 70.3% and 72.6% respectively. Held et al. (83) re-irradiated 124 ACC, in a cohort of 229 patients (54%) with recurrent head and neck cancers. The median reirradiation dose was 51 Gy(RBE). Median PFS and OS were respectively 24.2 months and 26.1 months. Hayashi (84) evaluated CIRT reirradiation on 17 ACC on a cohort of 48 patients with recurrent head and neck tumors. The median reirradiation doses ranged between 40.0 Gy(RBE) and 64 Gy(RBE) in 8 to 16 fractions. The median follow-up was 27.1 months; two-year local control, locoregional control, PFS and OS were 40.5%, 33.5%, 29.4% and 59.6% respectively. There was 10.4% of acute grade 3 toxicity and 37.5% of late grade 3 toxicity, including one grade 5 central nervous system necrosis. Finally, Vischioni et al. (85) evaluated CIRT re-irradiation on 38 ACC in a cohort of 51 patients (75%). The median prescription dose was 60 Gy(RBE). The median follow-up was 19 months; two-year PFS and OS were 52.2% and 64%. Two other studies have CIRT for head and neck recurrent tumor re-irradiation, but ACC histologies only represented a minority of the included cases: Combs et al. (77), who treated four ACC on a cohort of 28 patients, and Gao et al. (78) with 10 ACC on a cohort of 141 patients.

#### 4.2.4 Thoracic, Abdomen and Pelvis

Chen et al. (86) treated 18 patients with tracheobronchial adenoid cystic carcinoma with definitive CIRT treatment. Prescription doses ranged between 66 Gy(RBE) and 72 Gy(RBE). With a median FU of 20.7 months, the overall response rate (ORR) was 88.2%, and the 2-year OS, local control rate, and PFS were respectively 100%, 100%, and 61.4%. One G4 tracheal stenosis was observed, but no other grade 3 toxicity. Högerle et al. (79) treated six patients with tracheal ACC with CIRT (four patients received CIRT as a targeted boost added to photon irradiation); one patient was treated in an adjuvant setting, while six patients received definitive CIRT treatment. Prescription doses ranged between 60 Gy(RBE) and 74.4 Gy(RBE). One-year OS, freedom from local progression, and freedom from distant progression were both 100%. One grade 4 stomatitis was observed, but no other G3 toxicity. CIRT has also been proposed for gynecological ACC (87), but available clinical data is limited; to this date, only one patient seems to have been treated for a Bartholin’s gland ACC, with a 24 Gy(RBE) CIRT boost added to IMRT (50 Gy) (88).

### 4.3 Present and Future Considerations for CIRT Practice

#### 4.3.1 Treatment Planning System Considerations

Molinelli et al. (89) evaluated the possible prescription corrections in CIRT planning when comparing the modified microdosimetric kinetic model (mMKM) and the local effect model (LEM) relative biological effectiveness (RBE) models; overall, a 64 Gy(RBE) prescribed to the target volumes based on the mMKM model was found to be close to that of 68.8 Gy(RBE) with the LEM model. A new calculation of the OAR dose constraints, adapted to the RBE calculation model was therefore recommended in order to enhance the target control probability, such as for brain stem and optic pathway (90, 91).

For a full CIRT irradiation, ACC is generally irradiated with a sequential strategy consisting of a first phase of nine to ten fractions to a low-risk volume (including the surgical bed and zones at risk of perineural spread), followed by a second phase of six to seven fractions to a high risk volume (boost), with a unique nominal dose per fraction, according to the protocol adopted in Japan since 1997 (67). In this context, CIRT is usually delivered with a limited number of beams, typically two or three, achieving both a high conformation and an improved normal tissue sparing. However, in the absence of isocentric CIRT gantry, only fixed beam irradiations are available (which is for instance the case at CNAO) and it is difficult to change the beam arrangement between the two sequential phases: as a consequence, part of the low-risk volume receives unintended dose from the beam paths of the boost phase. A simultaneous integrated boost (SIB) approach is thus being evaluated at CNAO, in comparison to the sequential protocol, to improve the dose distribution of the two target volumes.

Robust planning is of prime importance for CIRT, to take into account range and positioning uncertainties (92). Finally, the use of Monte-Carlo algorithms combined with GPU-running processors make it possible to carry out extremely fast calculations, thus allowing the combination of accurate physical dose computation with biological effects modelling as well as inverse planning for CIRT, with a reasonable calculation time (93).

#### 4.3.2 Monitoring Tumoral Response and Anatomical Changes

The predicted range of carbon ion beams should be calculated as precisely as possible during treatment planning and treatment delivery. While small errors in margins quantification with photon RT leads to target underdosage, such errors with CIRT may have critical consequences; due to the sharp dose diminution at the distal edge of the Bragg peak, parts of the tumor might not receive any dose. The location of the distal dose fall-off notably depends on anatomical modifications, and minor anatomical changes might consequently negatively affect target coverage as well as substantially increase OAR toxicity. For head and neck ACC irradiation, where multiple nearby OARs have critical functions, CIRT might thus benefit from advanced techniques to monitor tumor response and potential anatomical changes during irradiation. This could for example be conveniently done by systematic CT scan reevaluations, the implementation of image guided CIRT, or robust planning. The dosimetric interest of adaptative planning for CIRT, relying on daily CT imaging, has been demonstrated for pancreatic irradiation (94) and might be conveniently implemented in the near future in clinical practice for hadrontherapy, possibly relying on automatic tools (95, 96). Fiorina et al. (97) demonstrated that in-beam positron emission tomography (PET) imageries allowed detection of morphological changes for head and neck proton therapy in clinical practice; while the primary fluence is lower in CIRT which introduces additional uncertainties compared to proton treatments, such devices have been successfully evaluated in phantoms for CIRT (98, 99) with a 1mm to 2mm agreement on the range prediction.

## 5 Discussion

We have detailed the rationale to use CIRT for ACC management, based on immunological, molecular, and pathological considerations, even though no *in vitro* or preclinical study have specifically evaluated CIRT irradiation on ACC cell lines to date. In addition, clinical data demonstrate that CIRT is associated with superior local control compared to conventional photon radiotherapy. In the future, further improvement of the outcomes of ACC treatment with CIRT may be possible by personalizing the treatment by taking into account ACC molecular and pathological features. Vered et al. (100) found that around 85% of ACC expressed EGFR receptors, which motivated the use of anti-EGFR systemic therapies in addition to radiation therapy, as radiosensitizers and to increase micrometastatic disease control. Adeberg et al. (101) recently evaluated adjunction of Cetuximab to photon radiation therapy (54 Gy) with a CIRT boost [24 Gy(RBE)], in 33 head and neck ACC patients. The toxicity was noticeable since 17% of the patients developed grade 3 rashes, 22% grade 3 radiation dermatitis and 48% grade 3 mucositis, but tumoral control was encouraging with a three-year DFS and OS of 67% and 90%, respectively. The ongoing NCT02942693 trial is a 2-arm study evaluating six weeks of Apatinib, an anti-VEGFR2 drug, followed by mixed irradiation with 56 Gy in photons and 15 Gy(RBE) in carbons. Immunotherapy combined with CIRT, although potentially attractive, considering the immunomodulatory effects of CIRT, is not currently evaluated in clinical trials.

However, CIRT might face competition with low-LET RT techniques for ACC management in the future. Takagi et al. (102) evaluated the outcome of 40 ACC patients treated with proton beam therapy (PBT) and 40 patients treated with CIRT; no difference between PBT and CIRT were observed in terms of OS, PFS, or local control at 5 years. However, in addition to the inherent weakness of a retrospective non-randomized comparison, there was a significant difference in the equivalent dose prescription between treatment groups in favor of PBT. Patient selection for CIRT (instead of PBT) must consequently be defined; NTCP or dosimetric considerations could be used. In some specific cases, fear of adverse events in case of rapid tumor shrinkage second to CIRT irradiation might justify considering normofractionated PBT irradiation. Reirradiation with photon stereotactic radiotherapy has been evaluated for relapsed head and neck ACC patients (103), with a total dose of 30 Gy in 5 fractions: the 3-year LC of 49% was lower than that observed with CIRT (possibly due to the relatively low prescribed dose), but this technique might be easily implemented in radiotherapy centers where hadrontherapy is not available. Other ongoing trials comparing CIRT with low-LET RT techniques include the ETOILE trial (NCT02838602) comparing CIRT and PBT or photon radiotherapy for radioresistant tumors, including ACC, and the COSMIC trial (NCT04214366) comparing CIRT with a mix of CIRT and photon beam radiotherapy for ACC. In addition, financial sustainability is an issue that may weigh against CIRT compared with other low-LET techniques. Jensen et al. (104) estimated that IMRT with a carbon boost might have a mean survival benefit of 0.86 years for a single head and neck ACC, with an incremental cost-effectiveness ratio of 20.638€ per year.

Finally, other high-LET techniques are currently being investigated for ACC, such as boron neutron capture therapy (BNCT), currently under development in various European and Asian centers. Kato et al. (105) treated one ACC patient to a dose of 14Gy, which was well-tolerated without any grade ≥2 toxicity. Other reports include Kankaanranta et al. (106), who re-irradiated four inoperable recurrent ACC and Aihara et al. (107), who treated four inoperable head and neck ACC (including two recurrent diseases). This latter reported complete response for all patients within 6 months, a median OS of 32 months and no grade ≥3 toxicity.

## 6 Conclusion

The use of CIRT for ACC management is motivated by immunological, molecular and clinicopathological considerations. Although no prospective randomized trials have been published to this date and might not be easily feasible, due to the rarity of ACCs and the scarce availability of particle beam RT facilities, clinical studies demonstrated that CIRT was well tolerated and associated with a substantial tumor control in diverse clinical situations, especially in advanced unresectable stages. Current technical developments ensure safe treatments.

## Author Contributions

Conceptualization: PL, BV, YK, and EO. Methodology: PL, BV, YK, and EO. Writing: PL. Review and Editing: PL, BV, MB, RI, SR, VV, AB, LG, LM, RD, RP, LL, VC, HM, SC, YK, and EO. Supervision: BV, YK, and EO. All authors contributed to the article and approved the submitted version.

## Conflict of Interest

The authors declare that the research was conducted in the absence of any commercial or financial relationships that could be construed as a potential conflict of interest.

## Publisher’s Note

All claims expressed in this article are solely those of the authors and do not necessarily represent those of their affiliated organizations, or those of the publisher, the editors and the reviewers. Any product that may be evaluated in this article, or claim that may be made by its manufacturer, is not guaranteed or endorsed by the publisher.

## Abbreviations

ACC, Adenoid cystic carcinoma; CD, Cluster of differentiation; CIRT, Carbon ion radiation therapy; CSC, Cancer stem cell; CTA, Cancer-testis antigen; DC, Dendritic cell; EGFR, Epithelial growth factor receptor; EMT, Epithelial-mesenchymal transition; HN, Head and neck; IHC,Immunohistochemistry; IMRT, Intensity modulated radiation therapy; LC, Local control; LEM, Local effect model; LET, Linear energy transfer; MDSC, Myeloid-derived suppressor cells; mMKM, Modified microdosimetric kinetic model; OER, Oxygen enhancement ratio; OS, Overall survival; PBT, Proton beam therapy; PFS, Progression free survival; PNI, Perineural invasion; RBE, Relative biological effectiveness; RFS, Relapse free survival; RNA, Ribonucleic acid; RT, Radiation therapy; TAA, Tumor-associated antigen; TAM, Tumor-associated macrophages; TCD50, Tumor control dose 50; TF, Transcription factor; TMB, Tumor mutational burden; TME, Tumor microenvironment; TNA, Tumor-specific neo-antigens; VEGF, Vascular endothelial growth factor.
